# Supplementary material for: Plant and Soil Microbial Diversity Co-Regulate Ecosystem Multifunctionality during Desertification in a Temperate Grassland
Source: Plants (Basel). 2023 Oct 31;12(21):3743. doi: 10.3390/plants12213743 (PMC10649343; doi:10.3390/plants12213743)
Supplement: Supplementary file 1 [file plants-12-03743-s001.zip › plants-2621422-supplementary.pdf]

Supporting information

Plant and soil microbial diversity co-regulate ecosystem multifunctionality during desertification in a temperate grassland

Table S1. Dominant plant species at the different stages of grassland desertification.

Desertification intensity	Vegetation cover	Above-ground biomass
**Potential desertification (Natural grassland)**	*Stipa baicalensis*<br>*Poa sphondylodes*<br>*Leymus chinensis*<br>*Carex*<br>*Heteropappus altaicun*<br>*Galium verum*<br>*Cleistogenes serotina*<br>*Artemisia dracunculus*<br>*Serratula centauroides*<br>*Lychnis fulgens* Fisch<br>*Takhtajaniantha austriaca*<br>*Artemisia tanacetifolia*<br>*Pulsatilla chinensis*<br>*Thalictrum squarrosum*<br>*Allium tenuissimum*<br>*Potentilla bifurca*<br>*Thermopsis lanceolata R. Br.*<br>*Orostachys fimbriata*<br>*Artemisia frigida*	**~150-170 g m^{-2}**
**Light desertification**	*Poa sphondylodes*<br>*Leymus chinensis*<br>*Carex*<br>*Serratula centauroides*<br>*Lychnis fulgens* Fisch<br>*Artemisia dracunculus*<br>*Artemisia tanacetifolia*<br>*Cleistogenes serotina*	**~120-130 g m^{-2}**

	Carex pediformis<br>*Thalictrum squarrosum*<br>*Potentilla bifurca*<br>*Iris ventricosa*<br>*Pulsatilla chinensis*<br>*Orostachys fimbriata*<br>*Allium tenuissimum*<br>*Artemisia frigida*	
**Moderate desertification**	*Leymus chinensis*<br>*Stipa capillata*<br>*Carex*<br>*Potentilla acaulis L.*<br>*Cleistogenes serotina*<br>*Galium verum*<br>*Artemisia frigida*<br>*Potentilla bifurca*<br>*Allium tenuissimum*<br>*Potentilla verticillaris Stephan*	**~80-90 g m^{-2}**
**Heavy desertification**	*Cleistogenes squarrasa*<br>*Carex pediformis*<br>*Salsola collina*<br>*Chenopodium glaucum*<br>*Gueldenstaedtia verna*<br>*Corispermum acrocarpum*<br>*Salsola collina*<br>Allium mongolicum	**~40-50g m^{-2}**
**Severe desertification**	*Corispermum acrocarpum*<br>*Salsola collina*<br>*Lappula myosotis*<br>*Chenopodium glaucum*<br>*Allium mongolicum*<br>*Setaria viridis*	**~10-20 g m^{-2}**
**Very severe desertification**	*Setaria viridis*<br>*Chenopodium glaucum*<br>*Salsola collina*	**~2-5 g m^{-2}**

Table S2. The effects of desertification intensity on plant diversity and soil microbial diversity under the potential desertification (PD), light desertification (LD), moderate desertification (MD), heavy desertification (HD), severe desertification (SD), and very severe desertification (VSD).The mean and SE (standard error) correspond to Figure 1.

	Plant diversity	Microbial diversity (OTUs)				
	(richness)	BAC	FUN	ARC	AMF	EMF
PD	14.87±0.27[a]	288.42±2.42[a]	278.55±3.76[a]	20.49±1.51[a]	29.568±0.88[b]	1.19±0.04[a]
LD	13.00±0.28[b]	278.68±2.65[a]	251.57±2.36[b]	16.78±1.31[ab]	37.078±0.45[a]	0.64±0.02[b]
MD	10.73±0.3[c]	266.93±3.16[b]	239.28±2.90[b]	13.31±1.03[bc]	31.46±1.31[b]	0.25±0.02[c]
HD	8.07±0.33[d]	253.34±1.79[c]	211.75±5.25[c]	14.67±1.40[bc]	31.354±1.44[b]	-0.17±0.02[d]
SD	6.47±0.26[e]	254.20±1.76[c]	209.64±2.59[c]	11.17±0.88[c]	30.715±1.67[b]	-0.73±0.03[e]
VSD	6.67±0.25[e]	232.27±2.26[d]	153.05±5.33[d]	10.44±1.33[c]	31.867±0.68[b]	-1.17±0.03[f]

*Mean ± SE (standard error); Significant differences among desertification intensity are indicated by different lower case letters in the same column ($p < 0.05$).

Table S3. The effects of desertification intensity on soil property at the six levels: potential desertification (PD), light desertification (LD), moderate desertification (MD), heavy desertification (HD), severe desertification (SD), and very severe desertification (VSD).

	Soil PH	Soil water content	Soil clay content
PD	$7.23{\pm}0.02^{d}$	$10.15{\pm}0.29^{a}$	$4.23{\pm}0.04^{a}$
LD	$7.25{\pm}0.04^{d}$	$9.58{\pm}0.20^{a}$	$4.03{\pm}0.05^{a}$
MD	$7.45{\pm}0.02^{c}$	$7.63{\pm}0.12^{b}$	$3.68{\pm}0.07^{b}$
HD	$7.57{\pm}0.03^{bc}$	$6.65{\pm}0.17^{c}$	$3.22{\pm}0.11^{c}$
SD	$7.70{\pm}0.03^{ab}$	$5.24{\pm}0.08^{d}$	$2.91{\pm}0.06^{c}$
VSD	$7.83{\pm}0.06^{a}$	$4.57{\pm}0.10^{d}$	$2.26{\pm}0.10^{d}$

∗Mean ± SE (standard error); Significant differences among desertification intensity are indicated by different lower case letters in the same column ($p < 0.05$).

Table S4. The theoretical information on 15 ecosystem functions and their importance.

**Individual function**	**Importance**	**Ecosystem function**	**Measuring method**
Soil total C	Soil total C was often used as a good proxy of ecosystem C stock.	C stock	The total C level of soil was determined with an elemental analyzer.
Soil phosphatase mineralization activity	It provides important information on phosphorus cycling and nutrient dynamics, contributing to an understanding of phosphorus availability in ecosystems and improved nutrient management.		A common test substrate is p-nitrophenyl phosphate, which is hydrolyzed by acid phosphatase to produce yellow p-nitrobenzene. A spectrophotometer can be used to measure the change in absorbance of p-nitrobenzene.
Phospholipid fatty acid	PLFA concentration has been used as a powerful indicator of soil live microbial biomass, which acts as a good proxy of microbial activity.	Decomposition and nutrient cycling	Measurement of fatty acid content within the cell membranes of microorganisms in soil to infer microbial biomass.
Soil N mineralization rate	The key process of N cycle transforming organic into inorganic N, and also acts as a good proxy of microbial activity.		Potential N mineralization was assessed in the lab using an aerobic procedure.
N	N is an important indicator_ for assessing soil N cycling.		
P	P is an important indicator for assessing soil P cycling.		
K	K is an important indicator_ for assessing soil nutrient content.	Nutrient stocks	The soil nutrients were determined with an elemental analyzer.
Ca	Ca is an important indicator for assessing soil nutrient content.		
Mg	Mg is an important nutrient element in soil widely involved in plant uptake and growth.		

Na	Na has an effect on plant growth.		
Fe	Fe has an important impact on plant growth and soil quality.		
Mn	Mn plays a key role in the growth and metabolic processes of plants.		
Cu	Cu is important for plant growth and development.		
Arbuscular mycorrhizal fungal infection rate	AMFI is a proxy of the plant-microbial symbiosis.	Symbiosis	AMFI was calculated by the number of first and second-order roots colonized by fungi divided by the total number of roots examined.
Plant biomass	Plant biomass is one of the most important indicators for assessing plant growth, productivity and ecosystem function.	Ecosystem productivity	All plant tissue from each quadrat was harvested to determine the aboveground biomass of vascular plants. To quantify root biomass, a cylindrical root sampler was used to gather three soil cores in each quadrat.

Table S5. Clonbach's coefficient to test data reliability.

Reliability statistics		
Cronbach Alpha	Cloning of Bach Alpha based on standardized terms	Number of items
0.687	0.883	9

Table S6. KMO values and Bartlett's sphericity test were used to replace validity tests.

KMO and Bartlett's test		
KMO The number of sample fitness measures.		0.936
Bartlett's sphericity test	Approximate cardinality	1320.774
	Degree of freedom	36
	Significance	0

Table S7. Relationships between biodiversity and soil properties on EMF.

	BAC	FUN	ARC	PD	PH	SWC	SCC	SD	EMF	AMF
BAC	1									
FUN	0.888**	1								
ARC	0.603**	0.543**	1							
PD	0.809**	0.809**	0.534**	1						
PH	-0.763**	-0.772**	-0.500**	-0.760**	1					
SWC	0.805**	0.827**	0.530**	0.877**	-0.811**	1				
SCC	0.812**	0.841**	0.452**	0.804**	-0.770**	0.867**	1			
SD	-0.879**	-0.905**	-0.550**	-0.922**	0.836**	-0.944**	-0.909**	1		
EMF	0.896**	0.922**	0.585**	0.915**	-0.827**	0.929**	0.901**	-0.991**	1	
AMF	0.068	0.007	0.118	0.13	-0.137	0.117	0.034	-0.076	0.045	1

Table S8. Analysis of variance for effects of desertification, their interactive effects on plant diversity, fifteen ecosystem functions and EMF.

Plant diversity & Ecosystem functioning	Significance	
	F	*P*
Plant diversity	151.573	0
NMR	246.881	0
N	155.871	0
Ca	82.421	0
Cu	236.29	0
Fe	418.411	0
K	123.174	0
Mg	240.37	0
Mn	311.952	0
Na	111.572	0
P	241.137	0
C	493.212	0
Plant C	274.222	0
PLFA	307.519	0
PMA	216.872	0
AMFI	10.689	0

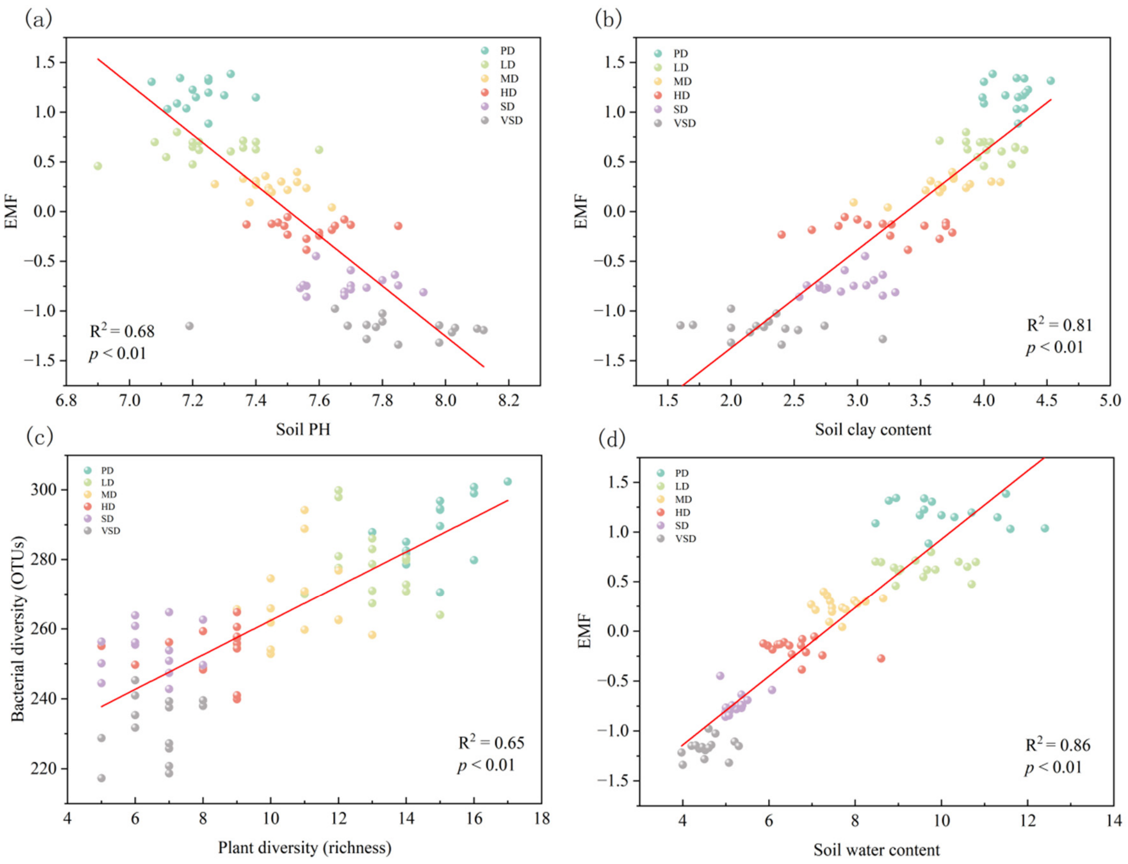

Figure S1. Relationships between EMF (a), soil water content (b), soil clay content (c), soil pH (d), and relationships between bacterial diversity (OTUs) and plant diversity (richness). The solid lines represent the fitted ordinary least squares.
